# The Aureochrome Photoreceptor PtAUREO1a Is a Highly Effective Blue Light Switch in Diatoms

**DOI:** 10.1016/j.isci.2020.101730

**Published:** 2020-10-24

**Authors:** Marcus Mann, Manuel Serif, Thomas Wrobel, Marion Eisenhut, Shvaita Madhuri, Samantha Flachbart, Andreas P.M. Weber, Bernard Lepetit, Christian Wilhelm, Peter G. Kroth

**Affiliations:** 1 Institut für Biologie, Universität Leipzig, 04009 Leipzig, Germany; 2 Fachbereich Biologie, Universität Konstanz, 78457 Konstanz, Germany; 3 Institut für Biochemie der Pflanzen, Cluster of Excellence on Plant Science (CEPLAS), Heinrich-Heine-Universität, 40225 Düsseldorf, Germany

**Keywords:** Photonics, Plant Genetics, Plant Biology, Transcriptomics

## Abstract

Aureochromes represent a unique type of blue light photoreceptors that possess a blue light sensing flavin-binding LOV-domain and a DNA-binding bZIP domain, thus being light-driven transcription factors. The diatom *Phaeodactylum tricornutum*, a member of the essential marine primary producers, possesses four aureochromes (PtAUREO1a, 1b, 1c, 2). Here we show a dramatic change in the global gene expression pattern of *P. tricornutum* wild-type cells after a shift from red to blue light. About 75% of the genes show significantly changed transcript levels already after 10 and 60 min of blue light exposure, which includes genes of major transcription factors as well as other photoreceptors. Very surprisingly, this light-induced regulation of gene expression is almost completely inhibited in independent PtAureo1a knockout lines. Such a massive and fast transcriptional change depending on one single photoreceptor is so far unprecedented. We conclude that PtAUREO1a plays a key role in diatoms upon blue light exposure.

## Highlights

• Blue light induces a very fast transcriptional response in the diatom *P. tricornutum*
• This strong response is almost completely inhibited when Aureochrome 1a is absent
• The results imply a key role of PtAureo1a in blue light-induced responses in diatoms

## Graphical Abstract

**Figure:**
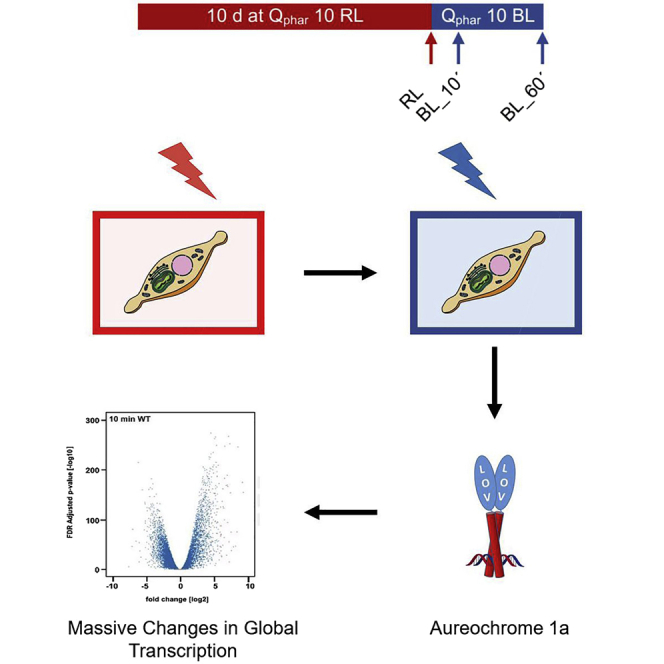

## Introduction

Diatoms are unicellular photoautotrophic algae that gained their plastids from secondary endosymbiosis (Archibald, 2015), in which a heterotrophic eukaryote engulfed a primitive unicellular red alga and converted it into a plastid (Cavalier-Smith, 2013). During this process, the resulting chimeric organism temporarily possessed five different genomes, two within the eukaryotic nuclei of host and endosymbiont, and three prokaryotic genomes within the mitochondria of the host and endosymbiont cell as well as one in the chloroplast. The nucleus and the mitochondria of the endosymbiont meanwhile have vanished in diatoms. Hence, a preceding massive gene transfer from the nucleus of the endosymbiont to the nucleus of the secondary host cell has to be assumed, leading to newly recombined genes and gene functions (Gruber, 2019; Nonoyama et al., 2019). Recent genome sequencing analyses showed that the diatom nuclear genome contains genes related to those of both plants and animals, including the green algal lineage, as well as a rather high percentage of bacterial genes due to lateral gene transfer (Bowler et al., 2008; Mock et al., 2017; Moustafa et al., 2009). In addition to their complex genome organization, diatoms differ in many aspects from green algae: Their cell walls consist of silica, the chloroplasts have a peculiar ultrastructure with four surrounding membranes, and the thylakoids are arranged in bands of three (Tanaka et al., 2015). Also, the basic pathways for energy conversion and carbon partitioning show a number of unusual features compared with higher plants and green or red algae (Bailleul et al., 2015; Flori et al., 2017). These genetic and physiological characteristics of diatoms are in line with an extremely fast evolution (Sims et al., 2006), leading to an estimated 100,000 diatom species (Adl et al., 2012). These evolutionary peculiarities coincide with an extraordinary ecological success: diatoms exist in nearly all aquatic and humid environments, encompassing oceans and freshwaters including their sediments, as well as soils, and aerophytic biofilms (Armbrust, 2009; Malviya et al., 2016). The diversity and environmental adaptability might be explained by the increased genetic background that may allow enhanced and unknown environmental acclimation strategies against a broad spectrum of stressors, e.g., light intensity (Lepetit et al., 2013; Wilhelm et al., 2014), UV-B (Beardall et al., 2009), as well as carbon dioxide and iron limitation (Goldman et al., 2019; Kolody et al., 2019).

For a photosynthetic organism, it is essential to determine and respond to changes in light quality and quantity. As land plants do, diatoms can sense light intensity via the activity of their photosystems and additionally possess photoreceptors that allow the sensing of both light intensity as well as wavelengths, including the diatom phytochromes (DPH) (Fortunato et al., 2016), different cryptochromes (cry) (König et al., 2017), heliorhodopsins (Pushkarev et al., 2018), and aureochromes (Kroth et al., 2017; Matiiv and Chekunova, 2018). Aureochromes have first been described in the siphonous xanthophyte *Vaucheria frigida* (Takahashi et al., 2007). They are very peculiar, as they are found in stramenopile algae only (Suetsugu and Wada, 2013), and they contain both a Flavin-binding LOV domain for blue light absorption and a bZIP domain for DNA binding (Takahashi et al., 2007). Thus, aureochromes essentially are light-responsive transcription factors and their simple topology makes them natural candidates for optogenetic tools in transcriptional regulation (Hepp et al., 2020). The common domain topology is inverted compared with all other characterized LOV proteins, with the sensory domain being located at the C terminus of the LOV receptor (Herman and Kottke, 2015). bZIP domains have a general tendency to dimerize, and only the dimeric bZIP domain is capable of binding DNA. For some aureochromes, dimerization and DNA binding have been shown to be induced by blue light (Banerjee et al., 2016b; Hisatomi et al., 2014; Nakatani and Hisatomi, 2015). However, the oligomerization state of full-length aureochromes is under debate. In the dark, PtAUREO1a has been observed as a monomer in equilibrium with a dimer (Heintz and Schlichting, 2016) or as a dimer/higher oligomer (Banerjee et al., 2016b). Light induces the dimerization of LOV domains and the association of the monomers (Herman et al., 2013; Goett-Zink et al., 2020; Kobayashi et al., 2020), which is the rate-limiting step in the process of DNA binding (Akiyama et al., 2016). The diatom *P. tricornutum* contains four genes encoding different aureochromes (Schellenberger Costa et al., 2013). Although all of them possess a LOV domain, only three of the respective gene products (PtAUREO1a/b/c) can actually bind a flavin, constituting the functional chromophore that allows detection of blue light (Banerjee et al., 2016b; Schellenberger Costa et al., 2013). The fourth aureochrome, PtAUREO2, carries a conserved mutation preventing flavin binding (Banerjee et al., 2016b), similarly to AUREO2 orthologs from other organisms.

Interestingly, the expression of some aureochromes follows a different diurnal pattern (Banerjee et al., 2016b), and there is evidence for a function of PtAUREO1a in cell cycle regulation (Huysman et al., 2013). Although previously created PtAureo1a silencing lines indicate an involvement of PtAUREO1a in triggering photosynthetic acclimation to different light colors and intensities (Schellenberger Costa et al., 2013), the influence of PtAUREO1a on the diatom transcriptome is yet unknown. It was the aim of this study to analyze the changes in the cellular transcriptome profile of the wild-type (WT) cells, PtAUREO1a-deficient mutants, as well as complemented lines of *P. tricornutum* after full acclimatization to red light and a subsequent shift to blue light. Importantly, for the light shift, we changed the wavelength, but not the number of photons absorbed by the photosystems, in order to exclude potential retrograde signaling effects induced by plastidic redox changes (Lepetit et al., 2013). Because it is known that in gene-silenced cell lines the amount of the investigated protein may be variable, we here chose to compare WT cells with two independent PtAureo1a knockout lines (Serif et al., 2017).

Our results demonstrate a dramatic change in the transcriptome upon shifting cells from red to blue light, affecting most cellular processes. This response is massively reduced in the PtAureo1a knockout lines, indicating a key role of the PtAUREO1a protein in cellular acclimation to blue light. As proteins that are known to be central in blue light responses in Archaeplastida, e.g., COP1 or HY5 (Podolec and Ulm, 2018), apparently are missing in diatoms, a fundamentally different regulation of blue light responses has to be assumed in diatoms and related algae.

## Results

### Dramatic Changes in *P. tricornutum* WT Cells When Shifted from Red to Blue Light

**Figure 1:**
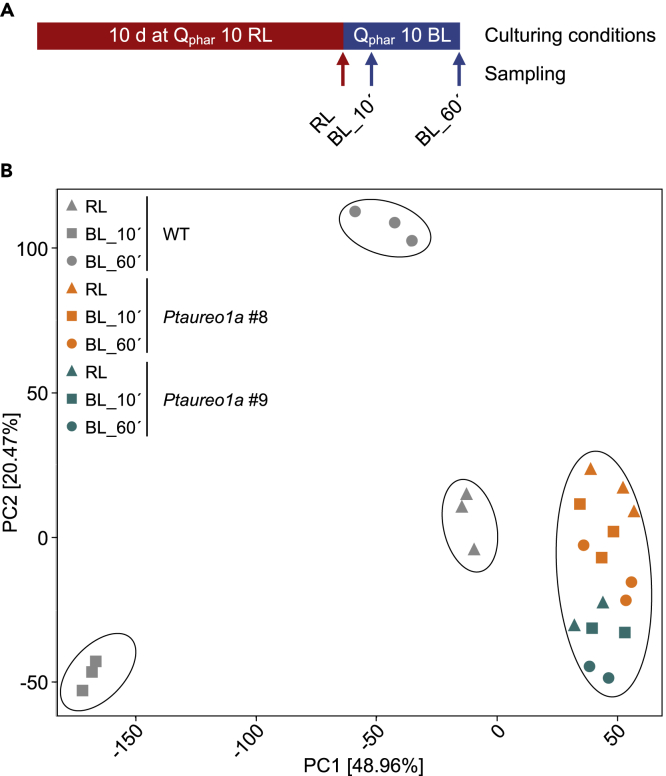
Transcriptomic Analyses of *P. tricornutum* Cells after a Shift from Red Light to Blue Light WT cells (three biological replicates), two independent Ptaureo1a mutant cells lines, *PtAureo1a #8* (three biological replicates) and *#9* (two biological replicates), were studied. (A) Scheme depicting the cultivation of the cell lines for 10 days at red light, followed by a shift to blue light comprising the identical amount of photosynthetically absorbed light quanta (Q_phar_). Samples were taken directly before the light shift (RL), and 10 (BL 10′) and 60 min (BL 60′) after the light shift. (B) Principal component analysis (PCA) shows initial changes for component 1 and long-term changes for component 2. WT samples in the same acclimation state, as well as all PtAureo1a mutant samples, are encircled.

In order to elucidate the impact of the transcription factor PtAUREO1a on the global expression pattern of *P. tricornutum* cells, we decided to monitor expression changes after a maximal change of the light trigger from red to blue light. We acclimated *P. tricornutum* WT cells (strain Pt4) for 10 days to red light, followed by a direct shift to blue light. As monochromatic red and blue light are differentially absorbed by the photosynthetic pigments in the diatom plastids, the light intensities were adjusted to an identical amount of photosynthetically absorbed light radiation (Qphar, calculated according to Gilbert et al., 2000) of 10 μmol photons m^−2^ s^−1^. We sampled in biological triplicates and generated transcriptomes that refer to the state directly before the shift as well as 10 and 60 min after the change of the light condition (Figure 1A) in order to observe the dynamics of this process. We had decided for the relatively short times of 10 and 60 min, because we expected that aureochromes as transcription factors may induce changes in gene transcription rapidly. Mapping of RNA-seq reads to the *P. tricornutum* genome (Phatr3, protists.ensembl.org/Phaeodactylum_tricornutum) identified 10,854 differentially expressed genes (87.6% of total genes). For the quantitative analyses reported here, only statistically significant changes of transcript abundance with a Benjamini-Hochberg corrected q-value of <0.01 were taken into account (Benjamini and Hochberg, 1995). In the WT, the principal component analysis (PCA) shows a dramatic change of total gene expression after the shift from red to blue light (Figure 1B). The three independent replicates of each time point consistently group together, whereas the samples of the individual time points show a clear separation (Figure 1B). The initial shift is covered in principal component 1 that explains 48.96% of the total variation, whereas the long-term reaction is mainly associated with principal component 2 that explains 20.47% of the total variation. This indicates that drastic changes in the gene expression pattern already occur 10 min after the shift to blue light, which differs strongly from the pattern at 60 min. Plotting probability values against log2-fold changes (Figure S1) indicates that the total number of upregulated genes is higher after 10 min, whereas the number of downregulated genes becomes higher after 60 min (both compared with the 0 min sample). After 10 min, about 74% of the genes are differentially regulated. About 60 min after the light shift, the number of non-regulated genes increased slightly to 39%, whereas about 30% of genes each are either up- or downregulated.

**Figure 2:**
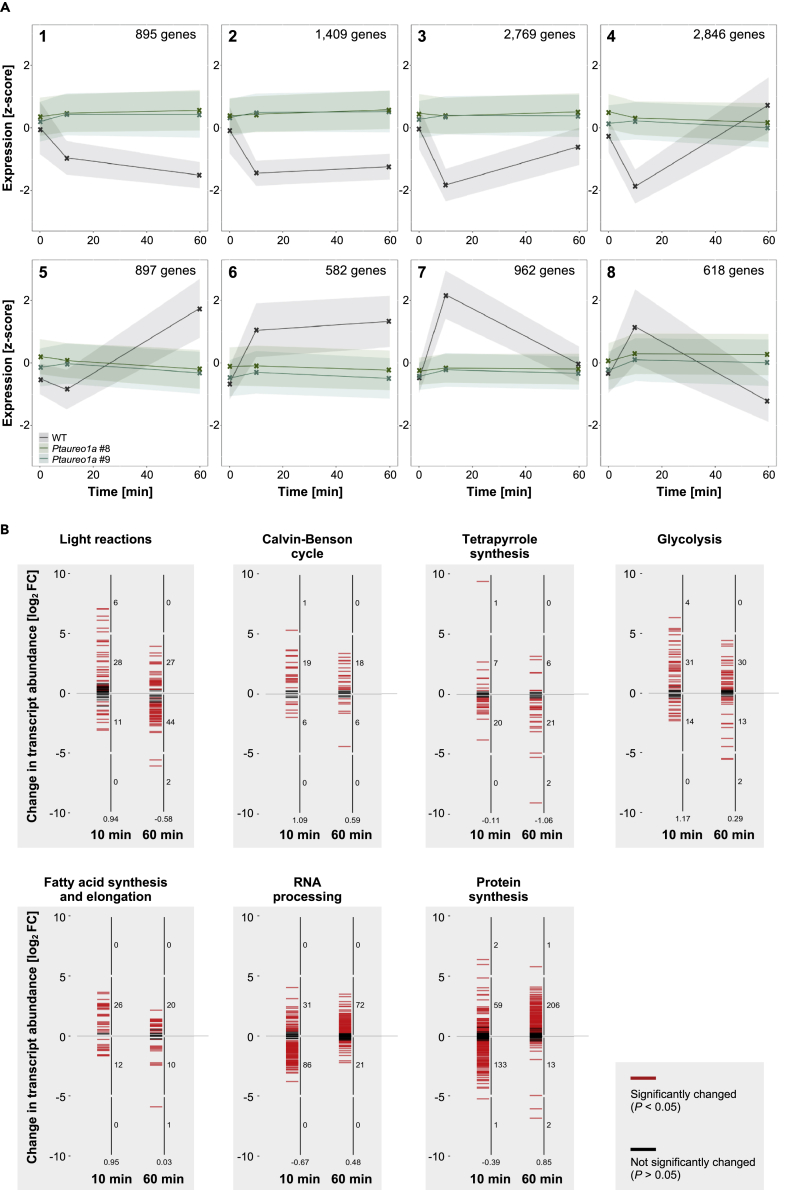
Clustering of Transcripts According to Blue Light Responses of Genes of Selected Pathways (A) We identified eight clusters that show different responses 10 and 60 min after a shift to blue light. The gray area indicates the range of transcript numbers of the WT cells, the green areas that of the two mutant cells lines. Where the area of both mutant lines overlaps, it is depicted in a darker green. (B) Clustering of up- and downregulated genes in WT cells in specific pathways that show a clear tendency. The y axis refers to log_2_-fold change at the respective time point compared with time point 0 (FC = fold change). The small numbers at the right side of the vertical axis indicate the number of genes regulated in the respective log_2_-fold interval covering five units, and the small number below the y axis refers to the average log_2_-fold change of all genes of this pathway.

In a hierarchical cluster analysis, we could identify eight clusters with different expression patterns (Figure 2A, gray lines): In clusters 1–5, gene expression decreases after 10 min, whereas clusters 6–8 show an increase of gene expression after 10 min. However, only in clusters 1,2, and 6 the increase/decrease is sustained after 60 min, whereas the other clusters return back to the original expression at t = 0 in a more or less strongly pronounced way or show the opposite response. Interestingly, clusters 4 and 5 show a strong decrease after 10 min, whereas, after 60 min, the expression values are higher than the starting point. The opposite behavior can be observed in cluster 8. When sorting genes according to gene ontology (GO), it becomes obvious that specifically the number of genes encoding enzymes of the light reaction, the Calvin-Benson cycle, glycolysis, and fatty acid metabolism, are strongly upregulated, especially after 10 min of blue light exposure. Instead, the majority of genes involved in tetrapyrrole synthesis is down-regulated (Figure 2B). Pathways that are involved in generation of proteins, e.g., genes encoding enzymes involved in RNA elongation and protein synthesis, interestingly show a strong down-regulation after 10 min, followed by an upregulation after 60 min.

In order to investigate how a shift to blue light may lead to such a quick response, we studied the expression of transcription factors more closely. A total of 214 transcription factors have been identified so far in the genome of *P. tricornutum* (Rayko et al., 2010; Matthijs et al., 2017), of which 210 could be detected and identified within our transcriptome dataset. Illumination with blue light results in a fast and pronounced response among these transcription factors: exposure to blue light for 10 min yielded a significant transcript increase of 40% and decrease of 42% of transcription factors, whereas after 60 min blue light exposure, 29% of transcription factor genes were upregulated and 33% were downregulated (Table 1). When the two different blue light samples (10 and 60 min) were compared with each other, it became apparent that these were in different adaptive states, with 33% of transcription factors upregulated and 44% downregulated after 60 min in comparison with the shorter blue light exposure. Thus, a short-term and a longer-term regulation can be seen in the WT cells, similarly to what was observed for the whole transcriptome. In order to identify groups of transcription factors that are more influenced by blue light exposure than others, we here examined the most prominent classes of transcription factors known in *P. tricornutum* in more detail. A total of 70 HSFs (heat shock factors), 40 zinc finger proteins, 34 Myb factors, 23 bZIP (basic leucine zipper domain) transcription factors, and 7 bHLH proteins together correspond to 81% of all transcription factors found in *P. tricornutum* (Rayko et al., 2010; Matthijs et al., 2017) (Table 1). In the WT cells, the shift from red to blue light led to a significant up- or downregulation of most of the members of these transcription factor classes after 10 min (see also Figure S2). The prolonged blue light treatment of 60 min resulted in more than 50% of transcripts of all major transcription factor classes to be up- or downregulated compared with the red light conditions. The large family of HSFs were substantially affected here with over 51% of transcripts downregulated and 17% upregulated at BL_60, respectively, whereas about 50% of the bZIP factors show an increased transcript abundance after 10 min.

**Table 1:** Effect of Blue Light Treatment on Transcript Levels of Transcription Factors

Transcription Factors	Regulation	WT_10 min_BL	WT_60 min_BL	WT_60 min/10 min_BL
All (214)	Up	86 (40%)	62 (29%)	71 (33%)
	Down	89 (42%)	70 (33%)	96 (44%)
HSF (70)	Up	26 (37%)	12 (17%)	18 (26%)
	Down	34 (49%)	36 (51%)	32 (71%)
Myb (34)	Up	9 (26%)	11 (32%)	17 (50%)
	Down	17 (50%)	7 (21%)	10 (29%)
bZIP (23)	Up	12 (52%)	4 (17%)	3 (13%)
	Down	6 (26%)	11 (48%)	17 (74%)
Zinc finger (40)	Up	17 (43%)	13 (33%)	17 (43%)
	Down	16 (40%)	9 (23%)	14 (35%)
bHLH (7)	Up	3 (43%)	3 (43%)	1 (14%)
	Down	2 (29%)	1 (14%)	5 (71%)

Given are numbers of significantly up-/down-regulated genes belonging to five important transcription factor classes found in *P. tricornutum* together with percentages with regard to all factors of that group (in parentheses). The total number of members of each group is given in parentheses (HSF, heat shock factors; Myb; bZIP, basic region leucine zipper; Zinc finger; bHLH, basic-helix-loop-helix).

### Differential Gene Expression in PtAureo1a Knockout Mutants

Previous studies revealed a regulatory function of PtAUREO1a in different processes such as high light acclimation and cell cycle (Huysman et al., 2013; Schellenberger Costa et al., 2013). In order to unravel the general function in blue light responses, we examined PtAureo1a knockout mutants in an identical experimental setting as described for the WT cells, including the same growth conditions and acclimation to red light. The knockout strains *Ptaureo1a#8* and *Ptaureo1a#9* had been created previously by genome editing via TALENs (Serif et al., 2017), and they have been characterized in detail to ensure that the PtAureo1a expression is abolished completely in both mutant lines (Serif et al., 2017). Moreover, the phenotypes of the mutants have been verified previously by complementation studies (Madhuri et al., 2019) to verify that in these mutants only the PtAureo1a gene had been affected. We observed that both PtAUREO1a-deficient strains, in contrast to the WT cells, show a very similar but weaker response to blue light. Figure 1B reveals that (1) the data points of *Ptaureo1a#8* (in biological triplicate samples) and *Ptaureo1a#9* (in duplicate samples) are located very close to each other, (2) the data points of the mutants all cluster close to the WT samples at time point 0, and (3) the samples of the individual mutants cluster closely together, irrespective of whether they were taken at time point 0, 10, or 60 min. This clearly indicates that the PtAureo1a knockout mutants respond very weakly to blue light, thus behaving very differently compared with the WT cells. This also becomes obvious when examining the response clusters (Figure 2A, green lines). In all clusters, the WT cells respond in one or the other way described above, whereas the mutant cell lines hardly respond at all.

We found that the two independent PtAureo1a knockout lines investigated here had very similar transcription profiles that are clearly distinct from those of the WT cells. We verified this result independently by qPCR-based gene expression analyses of two independent PtAureo1a complemented lines (Madhuri et al., 2019). We here focused on four genes, which exhibited a strong differential expression between WT and PtAureo1a knockout lines (UDP glucose pyrophosphorylase, HSF2, glycerol-3-phosphate dehydrogenase, and the gene Phatr3_J38559, see Figure S3). Although the culture conditions were not completely identical for technical reasons, the results confirm that the massive changes in gene expression upon a red to blue light shift, which are strongly dampened in the two PtAureo1a knockout lines, is re-established in the two PtAureo1a complemented lines to a similar extent as in the WT.

**Figure 3:**
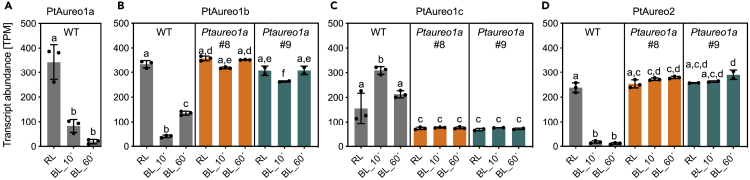
Transcript Abundance in TPM (Transcript per Million) of the Four Aureochrome Genes PtAureo1a/1b/1c/2 WT cells and two independent PtAureo1a knockout lines (*PtAureo1a #8* and *#9*) were studied (A: PtAureo1a; B: PtAureo1b; C: PtAureo1c; D: PtAureo2). For each gene, the average transcript abundance is shown for samples at time points 0, 10, 60 min after the light shift from red (RL) to blue (BL) light. Identical y axes have been chosen to allow a comparison of the transcript abundances. Statistics were performed using two-way ANOVA with Tukey test to correct for multiple comparisons. TPM values were called significantly different if the adjusted p value ≤0.05. Letters indicate statistical significance. TPM values sharing letters are not significantly different.

**Figure 4:**
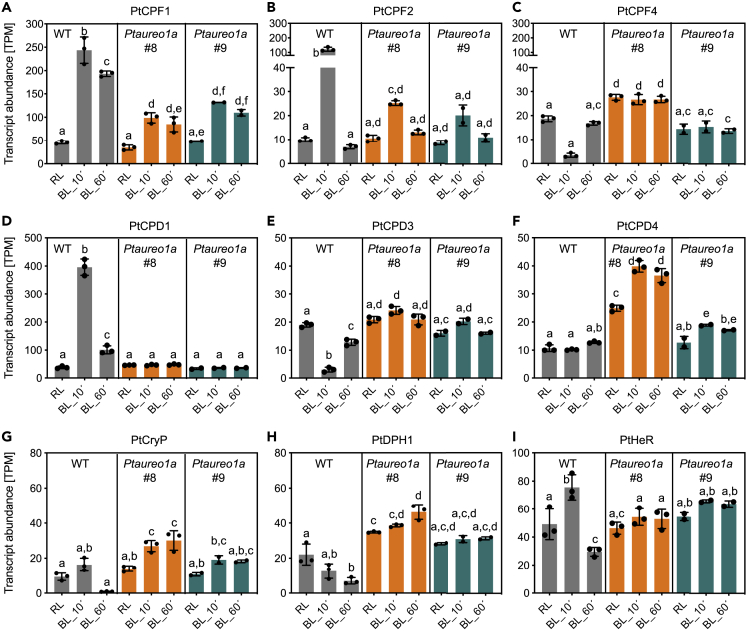
Transcript Abundance in TPM (Transcript per Million) of Genes of Diatom Photoreceptors and Related Proteins Transcript abundance of (A) PtCPF1, (B) PtCPF2, (C) PtCPF4 (cryptochrome photolyase family), (D) PtCPD1, (E) PtCPD3, (F) PtCPD4 (cyclobutane pyrimidine dimer photolyases), (G) the plant like cryptochrome PtCryP, (H) the diatom phytochrome PtDPH1, (I) the heliorhodopsin PtHeR in wild-type cells and in two independent PtAureo1a knockout lines (*PtAureo1a #8* and *#9*) are shown. For each gene, the average transcript abundance is shown for samples at time points 0, 10, 60 min after the light shift from red (RL) to blue (BL) light. Note that here different y axes have been chosen because of the large TPM differences. Statistics were performed using two-way ANOVA with Tukey test to correct for multiple comparisons. TPM values were called significantly different if the adjusted p value ≤0.05. Letters indicate statistical significance. TPM values sharing letters are not significantly different.

As we monitored the transcriptome during changes in light color, we specifically investigated gene expression regulation of PtAureo1a, 1b, 1c, 2 (Figure 3) and that of other diatom photoreceptors (Figure 4). Looking at the transcript abundances of PtAureo1a in the WT cells, we found that they are already comparably high at RL, whereas they are strongly reduced 10 min after the shift to blue light and close to zero after 60 min (Figure 3A). Similarly to PtAureo1a, the Ptaureo1b and PtAureo2 genes in WT cells are downregulated after blue light induction, whereas in the PtAureo1a knockout mutants, the transcript abundances of PtAureo1b and PtAureo2 are not affected by blue light, remaining on the previous red light level (Figures 3B and 3D). PtAureo1c shows the opposite response: transcription is induced by blue light, whereas in the mutant lines transcription remains unregulated on a very low level (Figure 3C). We also examined the response of transcript abundances of the other photoreceptors in *P. tricornutum*. Diatoms possess a number of cryptochrome-like proteins, for which it is not exactly known whether they primarily function in DNA repair or in blue light signaling (König et al., 2017). The cryptochrome/photolyase family proteins PtCPF1 and 2 are strongly upregulated by blue light in WT cells, while more weakly responding in the PtAureo1a KO mutants (Figures 4A and 4B). PtCPF4 transcripts are reduced after 10 min blue light in WT cells, while not responding in both PtAureo1a knockout mutants. The three cyclobutane pyrimidine dimer photolyases PtCPD1,3,4 show a different response (Figures 4D–4F): PtCPD1 is upregulated by blue light in WT cells, whereas the transcription of PtCPD3 is repressed and PtCPD4 instead is unaffected. All three genes, though, show a different mRNA pattern in the mutant lines, which is closer to the time point 0 in WT cells. The cryptochrome PtCryP shows a slight increase 10 min after the switch to blue light, whereas after 60 min, essentially no transcripts are detectable (Figure 4G). The red light-absorbing diatom phytochrome PtDPH1 shows inhibition of transcription by blue light in WT cells and no change in the knockout lines (Figure 4H). Finally, also the recently discovered heliorhodopsin PtHeR shows an impact of blue light on transcripts in the WT cells but not in the PtAureo1a knockout mutants (Figure 4I). Taken together, transcript amounts of all of these light-absorbing signaling proteins clearly show different dynamics in WT and in mutant lines, indicating that PtAUREO1a may modulate, directly or indirectly, their expression. Interestingly, most of the photoreceptors exhibited a comparable transcript level at time point 0 (thus in red light) in WT and KO mutants except for PtAureo1c (Figure 3C), which was lower in the mutants even at time point zero.

**Figure 5:**
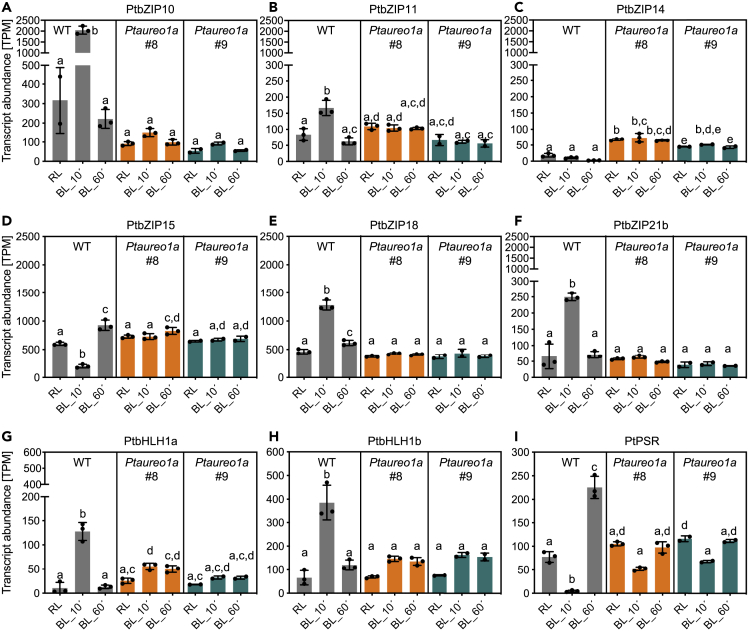
Transcript Abundance in TPM (Transcript per Million) of Genes of Selected Transcription Factors Transcript abundance of (A) PtbZIP10, (B) PtbZIP11, (C) PtbZIP14, (D) PtbZIP15, (E) PtbZIP18, (F) PtbZIP21b, the basic-helix-loop-helix transcription factors (G) PtbHLH1a (RITMO1) and (H) PtHlH1b, and (I) the Myb1R_SHAQKYF5 transcription factor (PtPSR) in wild-type cells and two independent PtAureo1a knockout lines (*PtAureo1a #8* and *#9*) are shown. For each gene, the average transcript abundance is shown for samples at time points 0, 10, 60 min after the light shift from red (RL) to blue (BL) light. Identical y axes have been chosen for two groups to allow a comparison of the transcript abundances. Statistics were performed using two-way ANOVA with Tukey test to correct for multiple comparisons. TPM values were called significantly different if adjusted p value ≤0.05. Letters indicate statistical significance. TPM values sharing letters are not significantly different.

As we had observed a massive regulation of transcription factors upon a red to blue light shift in the WT, we also compared the regulation of selected transcription factors in the PtAureo1a KO lines (Figure 5). We found very dramatic changes for PtbZIP10/11/18/21b, which all are strongly induced after 10 min blue light in the WT cells but which do not change much in the PtAureo1a mutants at the different time points. PtAUREO1a has previously been shown to be involved in both photoprotection and cell cycle progression after dark arrest (Huysman et al., 2013; Schellenberger Costa et al., 2013; Serif et al., 2017), the latter in cooperation with bZIP10 (Huysman et al., 2013). Interestingly, transcription of bZIP10 in WT is strongly upregulated upon blue light treatment for 10 min but downregulated after 60 min (and not induced in the mutant lines). Conversely, PtbZIP15 and the transcription factor PtPSR show a decreased transcript abundance after 10 min and a very strong increase after 60 min, which is not observed in the mutant lines. PtZIP14 instead shows a generally higher transcript abundance in the mutant lines. Overall, the studied bZIP transcription factors like PtbZIP10 and PtbZIP18 revealed a rather high transcript abundance. PtbHLH1a (RITMO1), which recently has been shown to be involved in the circadian regulation of *P. tricornutum* (Annunziata et al., 2019), is strongly transcribed after 10 min, and to a lesser extent after 60 min, which is mirrored much weaker in the mutant lines. Interestingly, PtbHLH1b, which is related to RITMO1, shows a very similar but even more pronounced expression pattern.

Finally, we also examined genes that are involved in post-translational modifications (PTMs) of histones like acetyl-transferases, deacetylases, and methyl-transferases (Figure S4). Interestingly, most of the genes encoding PTM-related proteins were less expressed in WT cells compared with PtAureo1a KO mutants at all time points. However, the most pronounced changes in transcription of PTM genes between WT cells and PtAureo1a KO were observed after 10 min, indicating that PtAureo1a is required for regulating the histone PTMs.

## Discussion

Photoreceptors are important for cellular acclimation, a dynamic process that includes sensing of environmental signals, and signaling to the nucleus in order to change gene expression, and cellular/organismic remodeling eventually. Light acclimation has been studied intensively in diatoms, showing that a shift from low to high light induces a reduction in light harvesting antenna size, and an increase of components of electron transport and C-assimilation, but also leads to higher activities of photoprotection, N-assimilation, protein biosynthesis, carbohydrate storage, nutrient transporters, and cell proliferation (Smith et al., 2019; Wilhelm et al., 2014). In green algae, the mechanism triggering this cellular reorganization is closely linked to the light-dependent redox state of the cells (reviewed in Burlacot et al., 2019). In diatoms, the redox state of the PQ pool is also involved in light acclimation reactions (Lepetit et al., 2013), but the redox control machinery in the plastid via the redox regulator thioredoxin is different from that of green algae (Matsuda and Kroth, 2014). Recently, it was shown that the light acclimation ability in diatoms instead is strongly dependent on the aureochrome photoreceptor system (Schellenberger Costa et al., 2013), which is lacking in the green lineage. PtbHLH1a (also called RITMO1) is involved in regulation of circadian rhythm of diatoms (Annunziata et al., 2019). Transcription of RITMO1 in our experiment is increased by about 30-fold upon blue light exposure for 10 min, suggesting that its rhythmicity might be triggered by activation of aureochromes and/or other photoreceptors. Especially for benthic diatoms, it has been shown that minor changes in their location within the sediments can already have a strong impact on wavelength-specific light attenuation (Cartaxana et al., 2016), requiring adequate responses. Aureochromes can affect gene expression directly, and such a direct interaction with promoters may result in faster responses to light changes compared with other photoreceptors that are relying on signal cascades that include, e.g., phosphorylation steps (Duanmu et al., 2017). We observed that blue light strongly influenced gene expression of genes of the Calvin cycle, the photosynthetic light reaction, the tetrapyrrole biosynthesis, and the lipid metabolism (Figure 2B). These processes are directly affected by available light intensity and color, as they may buffer a potential excess of light energy, for which blue light can be indicative. Indeed, in the PtAureo1a knockout mutants these responses are much weaker (see Table S3).

One important observation of this and previous work is that knocking out PtAUREO1a is not lethal but results in specific phenotypes like a reduced light acclimation and a drastically reduced blue light response. This indicates that the aureochromes in *P. tricornutum* may have specific functions, and, at least in case of PtAUREO1a, cannot complement each other. Instead, they may form a self-controlling network, similarly to land plant phytochromes and the respective PIF transcription factors (Legris et al., 2019). The expression and function of aureochromes in *P. tricornutum* can be regulated on different levels: First, the transcription of the aureochromes shows different diel patterns: the expression of PtAureo1a and 1c follows a diel cycle, whereas transcription of the PtAureo1b gene is light induced (Banerjee et al., 2016b). Second, we here observed that, in addition to this circadian control, PtAureo1b, 1c, and 2 show a different expression pattern in the PtAureo1a KO mutant lines: PtAureo1a, under blue light, has a negative impact on the expression of the PtAureo1b and 2 genes, whereas PtAureo1c transcription requires the presence and stimulation of PtAUREO1a (Figure 3). We further could show that all PtAureo genes are highly expressed under red light conditions in WT cells, whereas transcription of PtAureo1a, 1b, and 2 is strongly downregulated after 10 and 60 min of blue light exposure. This indicates that aureochromes themselves and—as the PtAureo genes are regulated in WT but not in PtAureo1a KO lines—apparently no other blue light receptor may inhibit the expression of these aureochromes or that red light is required for their transcription. PtAureo1c is the only aureochrome that shows an increased transcription after a change to blue light in WT cells. A third mode of aureochrome regulation is the potential formation of heterodimers with other aureochromes or possibly other bZIP transcription factors (Banerjee et al., 2016b). This makes a discussion on the exact regulatory role of PtAUREO1a more complicated, as we do not know yet, whether (1) the absence of PtAUREO1a homodimers in the mutant lines is responsible for the drastic decrease of blue light induction, or whether (2) other aureochromes or bZIP factors may require PtAUREO1a as partner for heterodimer formation. Indeed, hetero-dimerization of PtAUREO1a and PtAUREO1c has been demonstrated *in vitro* (Banerjee et al., 2016a; Essen et al., 2017).

Although some of the previous investigations on externally induced changes in gene expression in *P. tricornutum* focused on long-term acclimation (Cruz de Carvalho et al., 2016; Huang et al., 2019), others also took short-term changes into consideration. Bones and coworkers showed that *P. tricornutum* responds strongly to light exposure after 48 h in complete darkness, and also to equal doses of red, green, and blue light (Nymark et al., 2013; Valle et al., 2014). Although these experiments were performed via microarrays that cover only a number of pre-selected genes, thus giving limited insights into global gene expression, they demonstrate the strongest responses 30 min after the light shift, which is the earliest time point investigated in that work (Nymark et al., 2013). This clearly supports our hypothesis that the fast changes that we observed are most likely based on photoreceptors and a quick conversion of the response via transcription factors. Smith et al. (2019) studied the response of *P. tricornutum* to a shift from nitrogen to ammonium and found about 50% of all genes responding to N-deplete or N-replete conditions; binning of the responding genes resulted in 201 distinct clusters or response types. Although cluster selection can be done in different ways and to different extent, this number is much larger than the numbers of cluster that we obtained for the blue light response. This may indicate that nutrient responses are much less hierarchically initiated and conveyed than the responses to blue light that are transmitted via PtAureo1a.

So far, we have a very fragmented knowledge about aureochrome-mediated signaling processes. In land plants, photoreceptors lack DNA-binding motifs and therefore do not interact directly with promoters. Instead, complex light-induced developmental processes require interaction of the photoreceptors with key regulators like COP1/SPA and HY5, which either interact with other proteins or serve as transcription factors, multiplying the responses and controlling processes like photomorphogenesis (Hoecker, 2017; Podolec and Ulm, 2018). As such systems so far have not been identified in diatoms and other stramenopiles, these algae likely developed a different regulatory system that relies on aureochromes that are both photoreceptors and transcription factors. Genetic regulation in plants like *A. thaliana* depends much more on transcription factors than it may be the case in diatoms, which may reflect their higher morphological complexity. Although approximately 5% of the total genes in *A. thaliana* are estimated to be transcription factors (Riechmann et al., 2000), in *P. tricornutum* transcription factor genes only represent about 2% of its total genes (Rayko et al., 2010). Nonetheless, the 214 identified transcription factors (Rayko et al., 2010) in *P. tricornutum* allow an astonishingly and unprecedented strong response within a very short time frame of 10 min, majorly initiated by PtAUREO1a.

Given the huge number of affected genes including the drastic changes in transcript pattern of transcription factors, it seems though unlikely that the huge impact on cellular transcription, which is observed in the WT cells, but absent in the PtAureo1a knockout lines, is based on individual interaction of PtAUREO1a with all respective promoters. This notion is supported by our finding that PtAureo1a expression at time point 0 is not particularly high, compared with that of the other transcription factors (based on TPM values). It seems more likely that, besides a putative direct interaction of PtAUREO1a homodimers and heterodimers with promoters of target genes (see above), PtAUREO1a may be involved in changes of the expression of other transcription factors, which themselves are responsible for gene regulation (including that of other transcription factor genes). Such a cascade of transcription factor activities could explain the wide response by the limited number of PtAUREO photoreceptors, representing an equivalent to the signal cascades of other photoreceptors that are based on phosphorylation/dephosphorylation of signaling components, modulating gene transcription via more than one step. This described regulation may also occur in red light conditions, as we observed that transcripts of several transcription factors are virtually absent in PtAureo1a KO lines in red light, and therefore should not be able to influence gene expression already after 10 min of BL.

**Figure 6:**
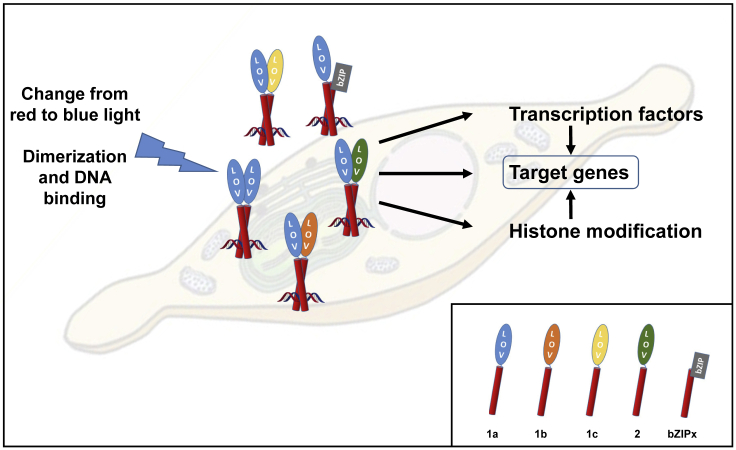
Hypothetical Model Describing the Impact of Aureochrome 1a (AUREO1a) on Gene Expression in *P. tricornutum* In the right lower corner, the individual aureochrome monomers and an exemplary bZIP protein are shown. PtAUREO1a, upon activation by blue light, may form homodimers, heterodimers with other aureochromes, or heterodimers with other bZIP proteins and bind to promoters. PtAUREO dimers may affect genes directly, and also via the transcriptional regulation of transcription factors, as well as by regulating the expression of histone modifying enzymes.

Besides the activity of transcription factors, the enzymatic modification of histones, for instance, by deacetylases and methylases, may have a strong impact on transcriptional activity. Such post-translational (PTM) activity has also been described recently for *P. tricornutum* (Veluchamy et al., 2013). As we could show that most of the genes known to be involved in PTMs are expressed to a lower degree in WT cells compared with PtAureo1a KO mutant lines, especially after the shift to blue light for 10 min (Figure S4), we hypothesize that histone modification may play an important role in PtAUREO1a-mediated responses (Figure 6).

In conclusion, it was very surprising to see that the deletion of a single blue light receptor (PtAUREO1a) has such a strong impact on gene expression, even though the cells possess several blue light receptors. This indicates that PtAUREO1a plays a central role in the perception of blue light by the algae. It may act on different regulatory levels, which include (1) the direct regulation of genes either as homo-dimers, aureochrome heterodimers, or heterodimers with other bZIP proteins like PtbZIP10 (Huysman et al., 2013), (2) the direct or indirect control of different transcription factors, and (3) the regulation of histone modifications. The aureochrome system of diatoms may serve as a future model to elucidate both top-down signaling cascades triggered by a single protein as well as diatom-specific regulatory mechanisms.

### Limitations of the Study

Here we show that diatom transcription responds dramatically to blue light and that mostly aureochrome 1a is responsible for this response. Aureochromes are only found in Stramenopile algae, which include different algal groups, like the ecologically important diatoms, chrysophytes, and brown algae. The results thus cannot be used directly to understand better the light regulation in land plants. We also have to proof experimentally in future analyses, which other transcription factors in which order are specifically involved in transmitting aureochrome-induced responses to such a large number of promoters.

### Resource Availability

#### Lead Contact

Further information and requests for resources should be directed to and will be fulfilled by the Lead Contact, Peter Kroth (peter.kroth@uni-konstanz.de).

#### Materials Availability

The strain used in this study (*P. tricornutum* Pt4, UTEX#646) is available from the UTEX Culture Collection (https://utex.org).

#### Data and Code Availability

The read data are deposited at the National Center for Biotechnology Information Gene Expression Omnibus (GEO) under accession number GSE158698. The datasets generated and analyzed during this study are available in the Supplemental Information.

## Methods

All methods can be found in the accompanying Transparent Methods supplemental file.

## Supplemental Information

Document S1. Transparent Methods, Figures S1–S4, and Table S1

Table S2. Differential Regulation of Transcription Factors Identified (A–C) Differential regulation of transcription factors according to Rayko et al., 2010 and Matthijs et al., 2017 upon 10 and 60 min blue light illumination in WT and PtAUREO1a knockout strains. Table S2A shows transcription factors sorted by family. Table S2B shows transcription factors primarily sorted by their regulation pattern after 10min of blue light illumination and secondarily sorted by TF family. Table S2C shows secondary sorting by log2-fold change of transcripts.

Table S3. Transcriptomic Data Based on Edge R Values, Related to Main Figure 2

Table S4. Transcriptomic Data Based on TMP Values, Related to Main Figure 2

## Footnotes

Supplemental Information can be found online at https://doi.org/10.1016/j.isci.2020.101730.
