# Supplementary material for: The Aureochrome Photoreceptor PtAUREO1a Is a Highly Effective Blue Light Switch in Diatoms
Source: iScience. 2020 Oct 24;23(11):101730. doi: 10.1016/j.isci.2020.101730 (PMC7670200; doi:10.1016/j.isci.2020.101730)
Supplement: Document S1. Transparent Methods, Figures S1–S4, and Table S1 [file mmc1.pdf]

iScience, Volume *23*

Supplemental Information

The Aureochrome Photoreceptor

PtAUREO1a Is a Highly Effective

Blue Light Switch in Diatoms

Marcus Mann, Manuel Serif, Thomas Wrobel, Marion Eisenhut, Shvaita Madhuri, Samantha Flachbart, Andreas P.M. Weber, Bernard Lepetit, Christian Wilhelm, and Peter G. Kroth

Figure S1: Volcano scatter plots displaying the probability (FDR adjusted p-value) against the log_2-fold change. **Related to Fig. 2.**

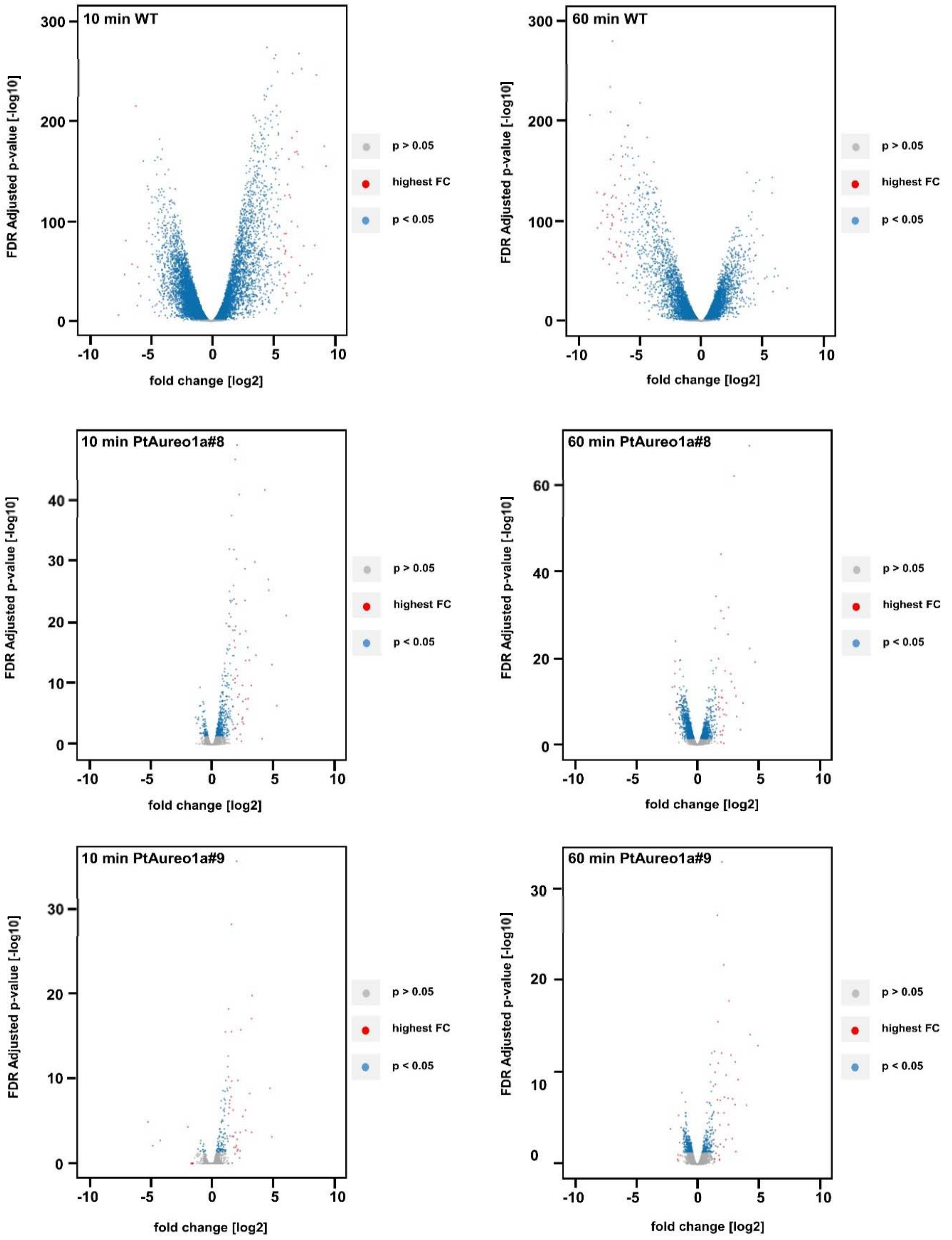

Figure S1: Volcano scatter plots displaying the probability (FDR adjusted p-value) against the log_2-fold change. In wild type cells, a large number of genes are either up- or down-regulated after a shift to 10 min of blue light, while after 60 min the majority of genes is down-regulated. Knockout mutants *PtAureo1a#8* und #9 show a similar pattern, but much weaker, as much less genes are regulated.

Figure S2: Clustering of up- and downregulated genes of groups of transcription factors from WT cells. Related to Fig. 5.

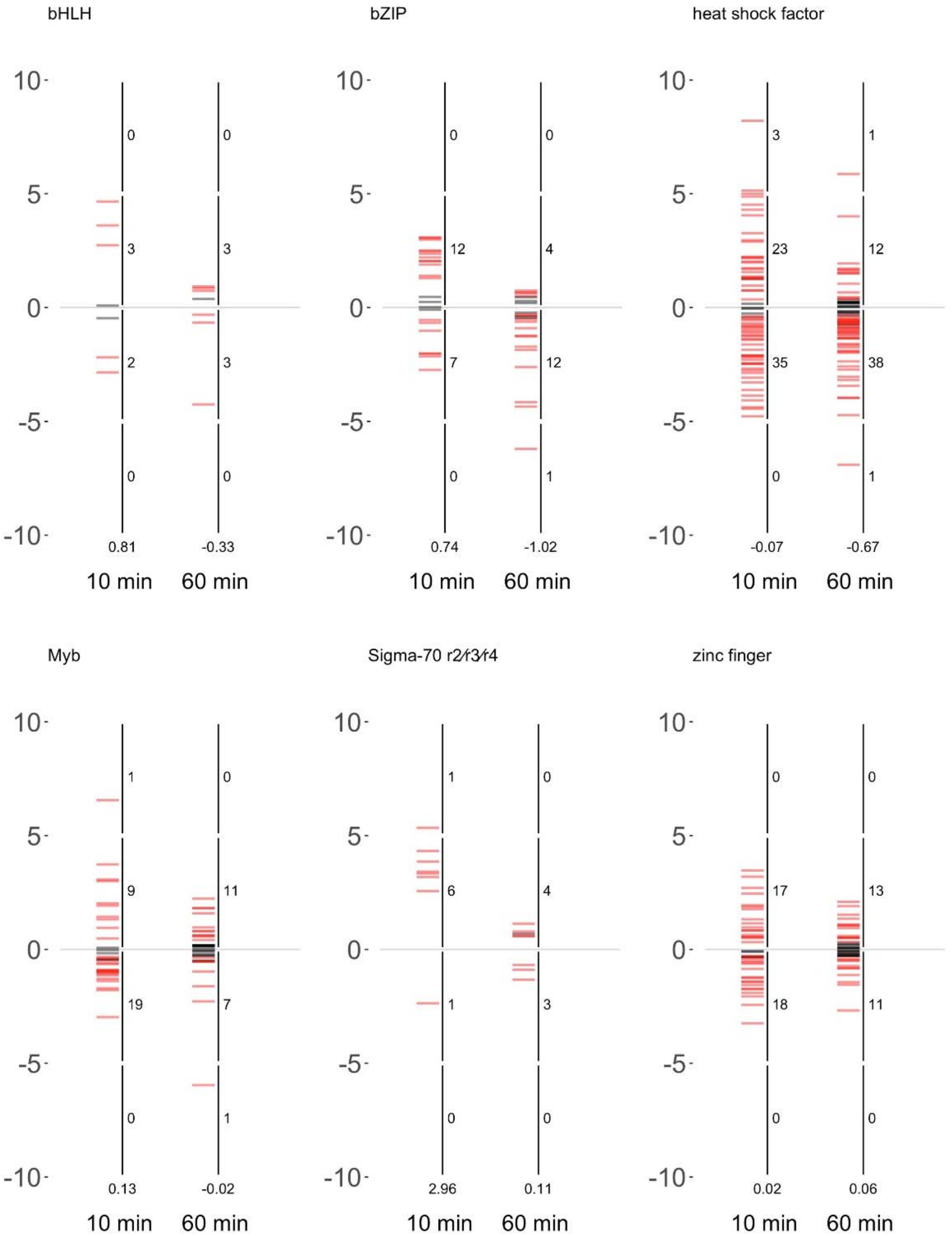

Figure S2: Clustering of up- and downregulated genes of groups of transcription factors from WT cells. The y-axis refers to log_2-fold change at the respective time point compared to time point 0, the small numbers at the right side of the vertical axis indicate the number of genes regulated in the respective log_2-fold interval covering five units and the small number below the y-axis refers to the average log_2-fold change of the whole transcription factor family. Red bars: significantly changes ($P<0.05$); black bars: not significantly changes ($P>0.5$). Most of the transcription factor families (with the exception of zinc finger proteins and heat shock factors) show more genes to be upregulated than downregulated after 10 min. After 60 min, most families show a balanced distribution or slight increase of downregulated genes (with the exception of zinc finger proteins).

Figure S3: Changes in expression of selected genes. Related to Transparent Methods.

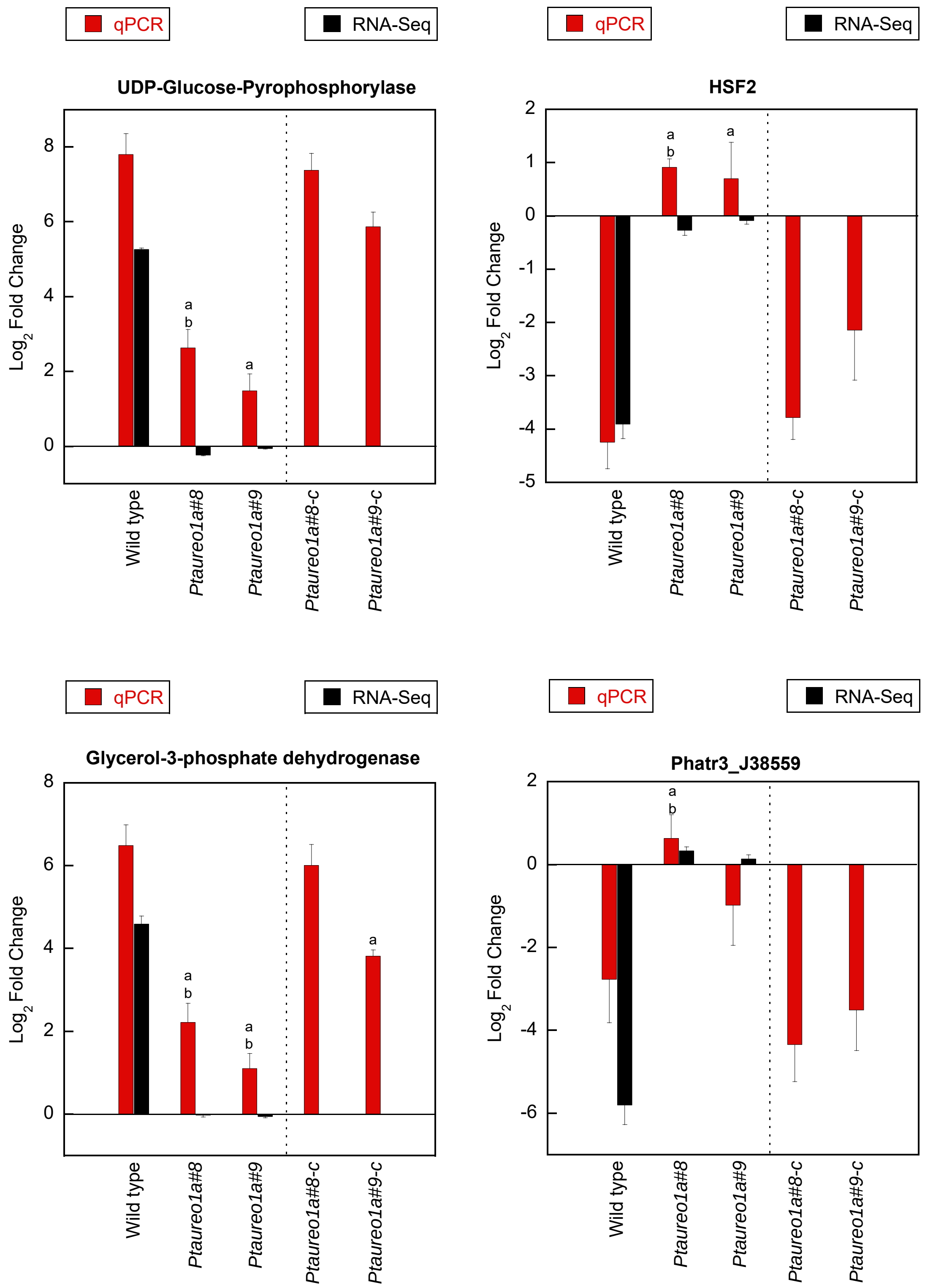

Figure S3: Changes in expression of selected genes (UDP glucose pyrophosporylase (Phatr3_EG02613), HSF2 (Phatr3_J55070), glycerol-3-phosphate dehydrogenase (Phatr_36821) and the undescribed gene Phatr3_J38559) after a shift from red light (28 µmol photons m^{-2} s^{-1}, measured with a LICOR planar quantum sensor) to 10 minutes of blue light (18 µmol photons m^{-2} s^{-1}), indicated in log_2-fold changes, in batch cultures of *P. tricornutum* WT, *Ptaureo1a#8, Ptaureo1a#9* and one complemented line for each knockout (*Ptaureo1a#8-c, Ptaureo1a#9-c*), determined by qPCR analyses of three biological replicates each. On the left side, genes that are upregulated upon a 10 min BL shift in the WT, based on the RNA-Seq analyses, are indicated. On the right side, genes that are downregulated upon a 10 min BL shift in WT cells, based on the RNA-Seq analyses, are indicated. For comparison, the log_2-fold changes for the respective genes, calculated from the TPM values of the RNA-Seq dataset, are indicated in black columns. Note that complemented lines have only been analyzed by qPCR and therefore are separated from WT and knockout lines by the dotted vertical line. Statistical significance for qPCR samples was determined using the Pairwise Fixed Reallocation Randomization Test performed by REST with 2000 randomizations. ‘a’ indicates statistical significance with $p<0.05$ compared to the WT, while ‘b’ indicates statistical significance of the knockout line compared to the respective complemented line with $p<0.05$. Standard error is indicated. A list of primers used is shown in Supplementary Table 1.

Figure S4: Log$_2$-fold transcript changes of putative histone modifying genes. Related to Fig. 6.

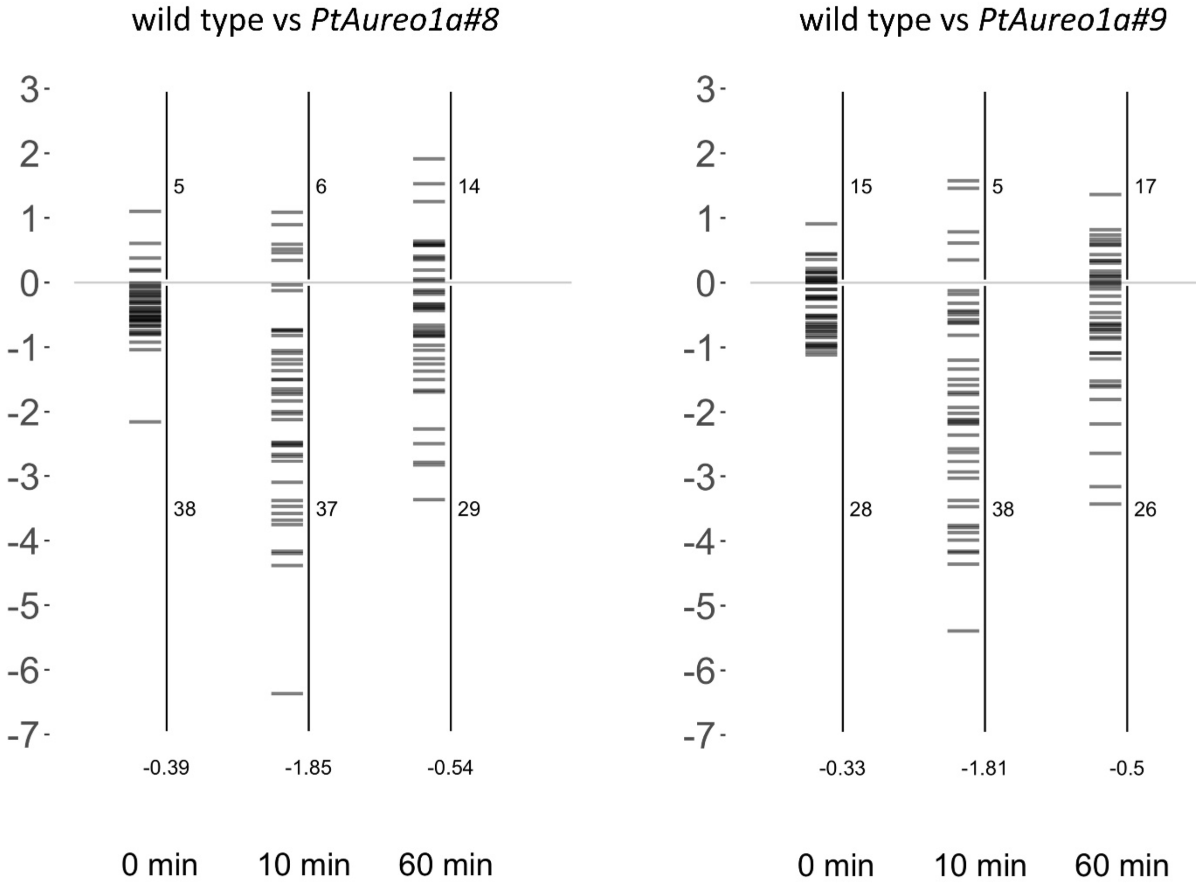

Figure S4: Log$_2$-fold transcript changes of putative histone modifying genes in WT vs *PtAureo1a#8* (a) and WT vs *PtAureo1a#9* (b) at time point 0, 10 and 60 min. The mean log$_2$-fold change for each gene is indicated as an individual bar. The numbers on the right side of the vertical axes indicate the amount of higher and lower expressed genes in the WT, respectively. The numbers below each plot indicate the average log$_2$-fold transcript change of all histone-modifying genes at the respective time point. The following genes have been included in the analyses (for details, see master tables: Supplemental Table 3/4 in individual files):

Phatr3_EG00952.t1, Phatr3_EG01307.t1, Phatr3_EG01943.t1, Phatr3_EG02126.t1, Phatr3_EG02442.t1, Phatr3_J1785.t1, Phatr3_J2957.t1, Phatr3_J3062.t1, Phatr3_J4423.t1, Phatr3_J4821.t1, Phatr3_J8891.t1, Phatr3_J9278.t1, Phatr3_J11358.t1, Phatr3_J35869.t1, Phatr3_J41559.t1, Phatr3_J42892.t1, Phatr3_J42944.t1, Phatr3_J43274.t1, Phatr3_J43627.t1, Phatr3_J43708.t1, Phatr3_J43919.t1, Phatr3_J44745.t1, Phatr3_J44757.t1, Phatr3_J44807.t1, Phatr3_J45431.t1, Phatr3_J45548.t1, Phatr3_J45703.t1, Phatr3_J45764.t1, Phatr3_J45906.t1, Phatr3_J46372.t1, Phatr3_J47425.t1, Phatr3_J49800.t1, Phatr3_J50465.t1, Phatr3_J50482.t1, Phatr3_J50848.t1, Phatr3_J51026.t1, Phatr3_J51040.t1, Phatr3_J51345.t1, Phatr3_J51406.t1, Phatr3_J54343.t1, Phatr3_J54505.t1, Phatr3_Jdraft1647.t1, Phatr3_Jdraft292.t1

Supplemental Table S1: **Primers used for qPCR analyses. Related to Figure S3.** The gene names refer to the Phaeodactylum genome database Phatr3 (https://protists.ensembl.org/Phaeodactylum_tricornutum/Info/Index)

Name	Forward primer sequence	Reverse primer sequence
UDP-Glucose-Pyrophosphorylase (Phatr3_EG02613) - fw	5'-GTCTCGTCTCGGGTGATTCC-3'	
UDP-Glucose-Pyrophosphorylase - rev		5'-CGTCCCCCTTGACTGGCAA-3'
Glycerol-3-phosphate dehydrogenase (Phatr_36821) - fw	5'-TCATCGGTAGCGGTAACTGG-3'	
Glycerol-3-phosphate dehydrogenase - rev		5'-ACCTGCGATTCGAAAAAGGG-3'
HSF2 (Phatr3_J55070) - fw	5'-ATGACTTACGCATCTCCCAATA-3'	
HSF2 - rev		5'-GGGTCGGTCAGTAGTTTTCGAG-3'
Phatr3_J38559 - fw	5'-TACTTGGAACGTCTCGGTCG-3'	
Phatr3_J38559 - rev		5'-TAGAAAGCGGTTGCCAAGAC-3'
Phatr3_J47943 - fw	5'-ATGCCGACACAGTCTACCG-3'	
Phatr3_J47943 - rev		5'-GGAGAGGTGGTTGTTGTTGC-3'

Transparent methods

Cultivation and harvesting of cells for RNA-Seq and qPCR

WT and PtAureo1a knockout strains K8 and K9 were cultured semi-continuously, in order to prevent nutrient limitation, as described in (Schellenberger Costa et al., 2013). The light intensity of blue and red light was adjusted to yield the same amount of photosynthetically absorbed radiation (Qphar 10 µmol photons m^{-2} s^{-1}) according to (Gilbert et al., 2000). Cells were adapted to a 14h/10h day night cycle with red light (28 µmol photons m^{-2} s^{-1}, 659 ± 11 nm) at 20°C for 10 days and then were shifted to blue light (18 µmol photons m^{-2} s^{-1}, 469 ± 10 nm). Samples were taken at time point t=0 (red light) as well as 10 min and 60 min of blue light: 50 ml of cells were filtered onto 1.2 µm RTTP polycarbonate filters (Merck-Millipore, Darmstadt, Germany), flash-frozen in liquid nitrogen and stored at -80°C until further use in RNA-Seq experiments. For verification of the transcriptome data, the expression of some selected genes in the WT line, the two PtAureo1a KO strains K8 and K9, and the two Aureo1a complemented lines Aureo1a8 Co56 (=*Ptaureo1a#8-c*) and Aureo1a9 Co59 (= *Ptaureo1a#9-c)* were analyzed via qPCR. The latter two strains are described and characterized in (Madhuri et al., 2019). The strains were cultivated in Erlenmeyer flasks on an orbital shaker in an Algaetron incubation chamber (PSI, Czech Republic) for 10 days at 20°C and 28 µmol photons m^{-2} s^{-1} of red light (636 nm) and then shifted to blue light (18 µmol photons m^{-2} s^{-1}, 440 nm) for 10 min.

Sample preparation for RNA-Seq

RNA was extracted from the filtered cells using the PeqGOLD RNApure and PeqGOLD RNA extraction kits (VWR, Darmstadt, Germany). Potential contamination with residual DNA was removed by an on-column digest with the PeqGOLD DNase I digest kit (VWR). Extracted RNA was quality-controlled with a spectrophotometer, by separation on an agarose gel, as well as on a 2100 bioanalyzer (Agilent, Waldbronn, Germany). cDNA libraries were prepared from 1 µg RNA using the TruSeqTM RNA Sample Prep Kit v2 (Illumina Inc., San Diego, USA). cDNA library generation and sequencing were performed with the Illumina HiSeq2000 in the paired end mode.

Bioinformatical analyses of the sequencing data and statistical information

Transcript abundances were determined by mapping the reads against the *P. tricornutum* CCAP 1055/1 genome (Release 27, downloaded June 14, 2015) in Ensembl

(http://protists.ensembl.org/Phaeodactylum_tricornutum/Info/Index, cDNA) using the CLC genomics workbench (https://www.qiagenbioinformatics.com/) with default parameters. Downstream analysis was performed in R version 3.6.1 (www.r-project.org). Differential gene expression was determined using edgeR (Robinson et al., 2009) on raw counts in GLM Fit mode with subsequent common and tagwise dispersion estimation. Principal component analyses and k-means clustering was performed on Z-scores calculated from TPM values. Samples were called significantly different if adjusted P value was <0.05. Gene enrichment for the k-means clusters was determined via the Fishers exact test using hand curated Mapman categories. To this end, the coding sequences from the *P. tricornutum* genome were compared to their *Arabidopsis* counterpart via bidirectional BLAST and the corresponding Mapman categories used as a starting point. BLAST results as well as the final Mapman categories are provided in Supplementary Tables 3 and 4. All P-values were Benjamini-Hochberg corrected before further analysis (Benjamini and Hochberg, 1995). The complete RNA-Seq data is also provided in Supplementary Tables 3 and 4. The read data are deposited at the National Center for Biotechnology Information Gene Expression Omnibus under accession number GSE158698. PCA analyses in Fig. 1 and the hierarchical cluster analyses were performed using the CLC genomics workbench software. For Figs. 3-5, TPM values of selected genes were plotted and statistically analyzed using GraphPad Prism version 8.4.2 (San Diego, CA). Two-way ANOVA with comparing every mean with every other mean was performed including Tukey test to correct for multiple comparisons.

Quantitative Real Time PCR (q-PCR)

RNA isolation for qPCR analysis was performed as described above. cDNA was synthesized using the Primescript kit (Takara Bio Europe, France). qPCR was performed with a 7500 Fast RT-PCR system (Applied Biosystems, USA) using a 2-step protocol. Each strain was measured in biological triplicates, and, on top, each gene per sample was measured in technical triplicates. Cycle threshold values and gene amplification efficiencies were obtained by utilizing PCR Miner 4.0 (Zhao and Fernald, 2005). Relative transcript levels were calculated according to (Pfaffl, 2001; Pfaffl et al., 2002). As 18S was not stably expressed under RL to BL shift conditions, we tested four genes which were stable during the shift and used one of them i.e. Phatr3_J47943.t1 (accession no. NC_011683) as reference gene. Primer sequences for all tested genes are supplied in Supplementary Table 1. Significance was tested using the Pairwise Fixed Reallocation Randomization Test performed by REST 2006 with 2000 randomizations (Pfaffl et al., 2002).

References for Supplemental Information

Benjamini, Y., and Hochberg, Y. (1995). Controlling the False Discovery Rate: A Practical and Powerful Approach to Multiple Testing. Journal of the Royal Statistical Society: Series B (Methodological) 57, 289-300.

Gilbert, M., Domin, A., Becker, A., and Wilhelm, C. (2000). Estimation of Primary Productivity by Chlorophyll a in vivo Fluorescence in Freshwater Phytoplankton. Photosynthetica 38, 111-126.

Madhuri, S., Río Bártulos, C., Serif, M., Lepetit, B., and Kroth, P.G. (2019). A strategy to complement PtAUREO1a in TALEN knockout strains of Phaeodactylum tricornutum. Algal Research 39, 101469.

Matthijs, M., Fabris, M., Obata, T., Foubert, I., Franco-Zorrilla, J.M., Solano, R., Fernie, A.R., Vyverman, W., and Goossens, A. (2017). The transcription factor bZIP14 regulates the TCA cycle in the diatom *Phaeodactylum tricornutum*. The EMBO Journal 36, 1559-1576.

Pfaffl, M.W. (2001). A new mathematical model for relative quantification in real-time RT-PCR. Nucleic Acids Research 29, e45.

Pfaffl, M.W., Horgan, G.W., and Dempfle, L. (2002). Relative expression software tool (REST©) for group-wise comparison and statistical analysis of relative expression results in real-time PCR. Nucleic Acids Research 30, e36-e36.

Rayko, E., Maumus, F., Maheswari, U., Jabbari, K., and Bowler, C. (2010). Transcription factor families inferred from genome sequences of photosynthetic stramenopiles. New Phytol 188, 52-66.

Robinson, M.D., McCarthy, D.J., and Smyth, G.K. (2009). edgeR: a Bioconductor package for differential expression analysis of digital gene expression data. Bioinformatics 26, 139-140.

Schellenberger Costa, B., Sachse, M., Jungandreas, A., Bartulos, C.R., Gruber, A., Jakob, T., Kroth, P.G., and Wilhelm, C. (2013). Aureochrome 1a is involved in the photoacclimation of the diatom *Phaeodactylum tricornutum*. PLOS ONE 8, e74451.

Zhao, S., and Fernald, R.D. (2005). Comprehensive Algorithm for Quantitative Real-Time Polymerase Chain Reaction. Journal of Computational Biology 12, 1047-1064.
